# Synthesis and Characterization of Citric Acid-Modified Iron Oxide Nanoparticles Prepared with Electrohydraulic Discharge Treatment

**DOI:** 10.3390/ma16020746

**Published:** 2023-01-12

**Authors:** Vladimer Mikelashvili, Shalva Kekutia, Jano Markhulia, Liana Saneblidze, Nino Maisuradze, Manfred Kriechbaum, László Almásy

**Affiliations:** 1 Nanocomposites Laboratory, Vladimer Chavchanidze Institute of Cybernetics of the Georgian Technical University, Z. Anjafaridze Str. 5, 0186 Tbilisi, Georgia; 2 Institute of Inorganic Chemistry, Graz University of Technology, Stremayrgasse 9/5, A-8010 Graz, Austria; 3 Research Institute for Energy Security and Environmental Safety, Centre for Energy Research, Konkoly-Thege Miklós Str. 29-33, 1121 Budapest, Hungary

**Keywords:** IONPs, SPIONs, biocompatible nanoparticles, citric acid capped iron oxide, SAXS

## Abstract

Chemical co-precipitation from ferrous and ferric salts at a 1:1.9 stoichiometric ratio in NH_4_OH base with ultrasonication (sonolysis) in a low vacuum environment has been used for obtaining colloidal suspensions of Fe_3_O_4_ nanoparticles coated with citric acid. Before coating, the nanoparticles were processed by electrohydraulic discharges with a high discharge current (several tens of amperes) in a water medium using a pulsed direct current. Magnetite nanoparticles were obtained with an average crystallite diameter D = 25–28 nm as obtained by XRD and particle sizes of 25 nm as measured by small-angle X-ray scattering. Magnetometry showed that all samples were superparamagnetic. The saturation magnetization for the citric acid covered samples after electrohydraulic processing showed higher value (58 emu/g) than for the directly coated samples (50 emu/g). Ultraviolet-visible spectroscopy and Fourier transform infrared spectroscopy showed the presence and binding of citric acid to the magnetite surface by chemisorption of carboxylate ions. Hydrodynamic sizes obtained from DLS and zeta potentials were 93 and 115 nm, −26 and −32 mV for the citric acid covered nanoparticles and 226 nm and 21 mV for the bare nanoparticles, respectively. The hydraulic discharge treatment resulted in a higher citric acid coverage and better particle dispersion. The developed method can be used in nanoparticle synthesis for biomedical applications.

## 1. Introduction

Nanomaterials in general, and nanoparticles (NPs) in particular are of great interest to researchers in various fields of science and technology. Because of their small size, high carrier capacity, high stability and the feasibility of incorporating them into both hydrophilic and hydrophobic substances, they are capable of different applications.

Among the various inorganic nanoparticles, iron oxide nanoparticles (IONPs) show unique physicochemical properties such as low Curie transition temperature and superparamagnetic nature; they are biocompatible, present low toxicity and show an antimicrobial activity [1,2,3,4,5]. Below a critical diameter, superparamagnetic iron oxide nanoparticles (SPIONs) possess a large constant magnetic moment which can be crucial for biomedical applications [6,7,8]. Their small size, which distinguishes them from bulk materials, yields a large surface-to-volume ratio; thus, NPs possess high surface energy which is beneficial for the functionalization of these nanoparticles with bioactive molecules as carriers of drugs, targeting molecules, and in general the creating the possibility of drug concentration by an external magnetic field

Nanosystems consisting of superparamagnetic magnetite (Fe_3_O_4_) NPs modified and stabilized with different kind of surfactant molecules and suspended in aqueous medium represent a versatile platform for both in vitro and in vivo applications such as drug delivery systems [9,10,11], hyperthermia [12,13,14], magnetic resonance imaging (MRI) [2,15,16] and magnetic labels for biosensing [17,18]. Besides biomedical applications, iron-based magnetic nanoparticles (MNPs) can be used in the fields of data storage [19], catalysis [20,21] and environmental remediation [22,23,24,25,26], and as plant protective agents [27,28] or plant growth stimulators [29,30]. Concerning their magnetic properties, iron oxide nanoparticles, in contrast to nanoparticles composed of transition metals such as Co, Ni and Mn, are favoured in biomedical applications because of the high toxicity of the latter compounds [31].

The usage of SPIONs has a great potential in the biomedical field. Their nano-scaled size and biocompatibility make MNPs compatible with cells. They mobilize within the blood stream, and due to their high magnetic moment, in the presence of an external magnetic field they can be targeted to pathologic tissues [32]. Successful applications of SPIONs rely on colloidal stabilization in an aqueous medium and precise control of shape, size, and size distribution that determine the physical and chemical properties of the nanocomposite. In particular, surface modifications are required to avoid agglomeration, as bare nanoparticles have insufficient long-term stability. They require hydrophilic and biocompatible surface coverage before they may be used in medical applications [33,34,35]. Alternative methods for synthesis and stabilization involve more elaborate procedures, such as a novel approach using microfluidics [36,37,38].

Surface coverage with citric acid (CA, C_6_H_8_O_7_) provides a thermodynamically stable colloidal solution [39]. Citric acid is a widely used organic coating material in the manufacture of nanoparticles since it can change surfaces’ charge and hydrophobicity, leaving additional carboxyl groups on the nanoparticle surface. CA shows bactericidal and bacteriostatic effects, and solutions containing citric acid are also used as sterilizing agents [40] and plant growth stimulators [41]. In addition, the biocompatibility and low toxicity of nanosystems consisting of water dispersed SPIONs (Fe_3_O_4_) modified with citric acid and serving as linker agents with anticancer drugs make them very interesting for biomedical applications, especially as drug delivery systems and MRI agents in modern healthcare [42].

A number of studies have been carried out on citric acid, as a widely accepted stabilizer material in water-based ferrofluids [39,43,44,45,46]. Adding CA during the chemical co-precipitation of ferrous salts allows one to control the size of the primary NPs and simultaneously prevent their aggregation [47]. Thus, through adding aqueous CA solution at different stages of synthesis, the core sizes of CA-capped IONPs could be adjusted in the range from 6 nm to 13 nm [48].

MNP synthesis and CA-functionalization in aqueous CA solution using co-participation at lower temperatures and a shortened time compared with conventional methods has been reported [49]. In a two-step process for synthesis, the addition of citric acid at decreasing coating temperatures resulted in increased hydrodynamic sizes of final product which also affect superparamagnetic feature of obtained material [44]. CA is believed to adsorb on the surface of nanoparticles in the monolayer, binding by coordinating ≡FeOH sites via one or two carboxylate groups depending on the steric necessity and surface curvature of NPs [50,51].

The application of electrohydraulic discharges (EHD) in water and organic liquids has been studied for many years. Electrical transmission processes initiate different types of chemical reactions and effects on the physical processes (e.g., degassing, decomposition, homogenization, cavitation, shock waves and ultraviolet/visible electromagnetic radiation), and promote a variety of chemical reactions [52]. A pulsed discharge can be advantageous as a pre-treatment stage before capping the CA, since the shock-wave effect on the suspension destroys the large agglomerates without significant destruction of the initial nanostructures. The efficiency of agglomerate dispersion by electrohydraulic discharge is higher than that of dispersion employing ultrasonication [33,53].

The aim of the present work is to explore the influence of high-voltage pulsed discharge (HVPD) on the stabilization quality of citric acid-coated SPIONs. To date, very few studies report investigations on the usage of electrohydraulic processing during the synthesis of magnetic nanofluids. In this work, we applied HVPD processing in the intermediate phase, before coating the iron oxide nanoparticle with the stabilizing CA molecule and revealing the effect of this treatment on the quality and properties of the resulting magnetic fluid.

## 2. Materials and Methods

### 2.1. Materials and Characterization Techniques

The chemicals used for the synthesis of magnetite nanoparticles were of analytical grade without further purification. Ferric chloride hexahydrate (FeCl_3_·6H_2_O) (≥98%), ferrous sulfate heptahydrate (FeSO_4_·7H_2_O), ammonium hydroxide solution (NH_4_OH, 25% of NH_3_ basis) and citric acid monohydrate (HOC(COOH)(CH_2_COOH)_2_·H_2_O)) ≥99.0% were purchased from Sigma-Aldrich Co. LLC (Darmstadt, Germany).

X-ray powder diffraction (XRD) analysis was performed using a DRON 3M X-ray diffractometer, operating with Cu Kα radiation (λ = 0.1541 nm) filtered by a nickel foil (voltage 40 kV, current 20 mA, and scanning speed 2°/min).

Magnetic measurements were performed on a vibrating sample magnetometer (VSM) (7300 Series VSM System, Lake Shore Cryotronics, Inc., Westerville, OH, USA) at room temperature under an applied field up to 1.5 Tesla.

Fourier transform infrared spectroscopy (FTIR) was performed on the Agilent Cary 630 FTIR spectrometer with 320-Cary FTIR Diamond ATR (spectral range: 6300–350 cm^−1^).

UV-Vis spectroscopy was performed using an AvaSpec-HS2048XL instrument with AvaLight-DHc light source allowing measurements in the 200–1160 nm spectral range. 

The hydrodynamic size distribution profile and zeta potential (ζ) of the aqueous suspensions were measured using Anton Paar Litesizer™ 500 equipped with a 658 nm laser, in backscattering geometry, thermostated at 25 °C, with a scattering angle of 173° and adjusted voltage 200 V.

Small-angle X-ray scattering (SAXS) was performed on a SAXSpoint 2.0 instrument (Anton Paar GmbH, Graz, Austria) equipped with a MicroSource Primux 100 copper X-ray generator (λ = 0.154 nm) and an Eiger R 1M position sensitive detector. Liquid samples were injected into quartz capillary and measured at 25 °C.

### 2.2. Synthesis Methods

#### 2.2.1. Synthesis of Bare Iron Oxide Nanoparticles

Bare (uncovered) iron oxide (Fe_3_O_4_) nanoparticles were prepared by sonochemical co-precipitation with ultrasound processing using an iron salt ratio Fe^3+/^Fe^2+^ of 1.9. First, FeCl_3_·6H_2_O (9 g) + 333 mL distilled water (DW) (0.1 M solution) was prepared in the jacketed reactor with mechanical stirring (temperature 45 °C, mixing duration 20 min, vacuum environment), and FeSO_4_·7H_2_O (4.87 g) +175 mL DW (0.1 M solution) in the jacketed ultrasonic reactor (temperature 45 °C, duration 20 min, ultrasonication 30% of 900 W homogenizer). After separate dissolution, the iron salt solutions were collected in an ultrasonic reactor and treated by ultrasonication and vacuum degassing for an additional 15 min. During this time the temperature was raised up to 55 °C, and previously prepared 19 mL NH_4_OH (25%) + 35 mL DW (4 M solution) was added dropwise over the course of 16 min by a peristaltic pump in the middle area of the reactor. After the completion of the supply of NH_4_OH solution, the sonication continued for additional 120 min without temperature control. The obtained black precipitate was cooled down to room temperature under ultrasonication.

In order to remove residues of the chemical synthesis and reduce the pH (initial pH 10 after synthesis) to the physiological value (pH 7.3), the particles were washed several times with an abundant quantity of DW with magnetic separation using a permanent magnet. After final washing, the vessel was filled up to 500 mL of DW and ultrasonicated with 30% of a 900 W power homogenizer for 30 min. A resulted suspension consisting of bare/uncovered MNPs was labelled bare-SPIONs. A total of 100 mL of this suspension was prepared, with a calculated maximum possible mass of the solid phase of 0.77 g (concentration 0.77 weight/volume percent).

#### 2.2.2. Electrohydraulic (EHD) Processing and Modification with Citric Acid

As in our previous study with folic acid conjugated IONPs, HVPD (electrohydraulic discharge) was applied before capping with CA in an aqueous medium as a surface activation and homogenization technique [33]. To modify the surface of MNPs with a carboxyl group, an aqueous solution of CA was added directly using a peristaltic pump, at atmospheric pressure and room temperature. Briefly, the 100 mL suspension was ultrasonicated with 30% of a 900 W power homogenizer over the course of 10 min, and previously prepared 0.19 g of CA (about 25% of magnetite) + 10 mL DW was dropwise added to the MNPs suspension over 10 min under ultrasonication (14% of 900 W power), followed by an additional 10 min of ultrasonication. 

The resulting sample’s (SPIONs-CA) pH was regulated using NH_4_OH aqueous solution until the pH reached 6 (initial pH 4.3), and the solution was stored for one night. On the following day, the SPIONs-CA solution was washed by DW to remove the excess of CA by decantation on permanent magnet, before being ultrasonicated again for 5 min.

**Figure 1:**
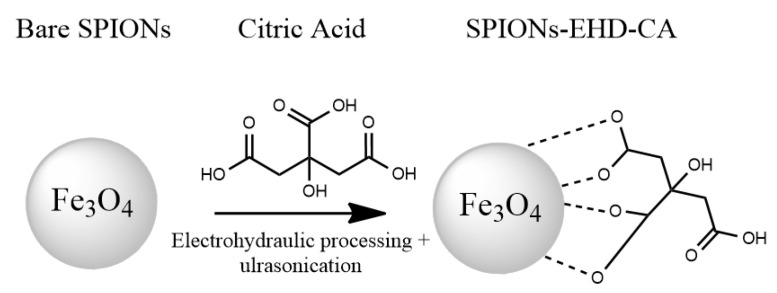
Scheme for preparation of magnetic nanoparticles by electrohydraulic discharges followed by modification with CA.

The SPIONs-EHD-CA sample was prepared in a similar way, with the difference that electrohydraulic treatment was applied on the bare-SPIONs suspension by pulsed arc discharges before capping the particles with CA (Figure 1).

The electrohydraulic processing was performed in the high current mode, as described in a previous work [33]. This mode allows low voltage and high current discharges inside the closed 300 mL volume reactor in a low vacuum environment (1 kPa). The distance between the electrode rods was d = 0.7 mm, the discharge peak current I_max_ = 30 A, voltage V = 1.2 kV, discharge frequency f = 2 Hz and the maximum impulse duration t_max_ = 20 ms. The experimental setup is described in [33].

## 3. Results and Discussion

### 3.1. X-ray Diffraction (XRD)

**Figure 2:**
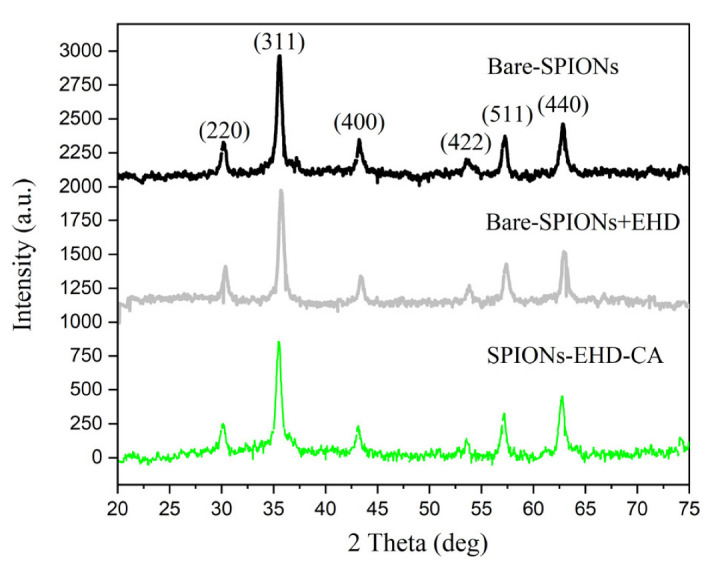
XRD patterns of synthesized bare-SPIONs, electrohydraulically processed bare-SPIONs and the CA-modified sample after EHD processing (SPIONs-EHD-CA). The data are shifted vertically for better visibility.

From the diffraction data of obtained samples, the diffraction peaks at 2θ values were assigned to the crystal planes (220), (311), (400), (422), (511) and (440), respectively (Figure 2).

All peaks match well with characteristic peaks of magnetite (Fe_3_O_4_) (JCPDS file no. 19-0629). There was no sign of any phase transition between electrohydraulically processed samples and the bare-SPIONs. Additionally, no crystalline impurity phases were observed. The average crystallite diameter (D = 28 ± 2 nm) was calculated using the Scherrer equation from the FWHM (full width at half maximum) of the (311) peak at 2θ = 35.86°. The average value of the lattice parameter was found to be a = 0.837 ± 0.001 nm.

### 3.2. Vibrating Sample Magnetometry (VSM)

**Figure 3:**
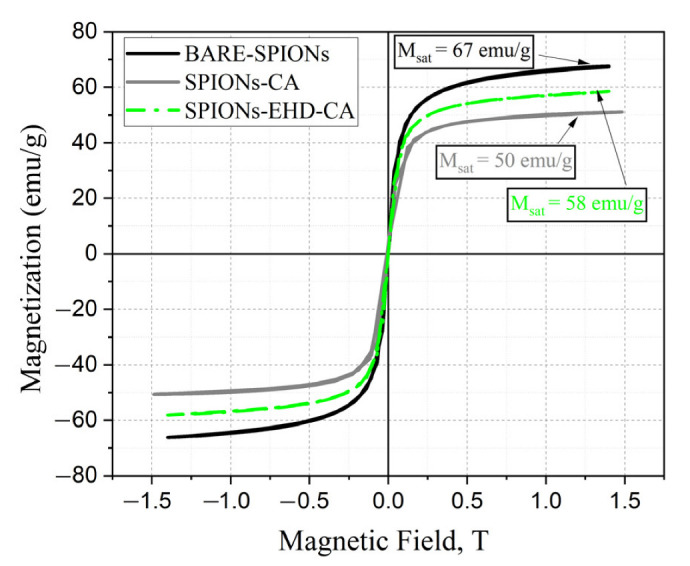
VSM results of synthesized IONPs with no coating (BARE-SPIONs), CA coated after electrohydraulic processing and directly coated with CA.

The magnetic properties of the obtained nanoparticles were investigated by VSM at room temperature (Figure 3). The hysteresis loops show the superparamagnetic behaviour of all samples.

The samples show no magnetic hysteresis; the magnetization and demagnetization on the curves pass through the origin, implying their superparamagnetic nature, which is an essential property of such nanoparticles in many applications. The bare-SPIONs exhibit higher magnetic saturation (M_sat_ = 67 emu/g) while the nonmagnetic CA reduces M_sat_ to 50 and 58 emu/g for the coated samples. This is the signature of the core–shell structure of the coated magnetite nanoparticles SPIONs-CA and SPIONs-EHD-CA, in which the weight percentage of Fe_3_O_4_ is lower.

The destruction of the aggregated bare nanoparticles formed after synthesis by the additional electrohydraulic discharge treatment resulted in a more efficient modification (shown by FTIR data next) and higher magnetization (58 emu/g).

### 3.3. Fourier-Transform Infrared Spectroscopy (FTIR)

**Figure 4:**
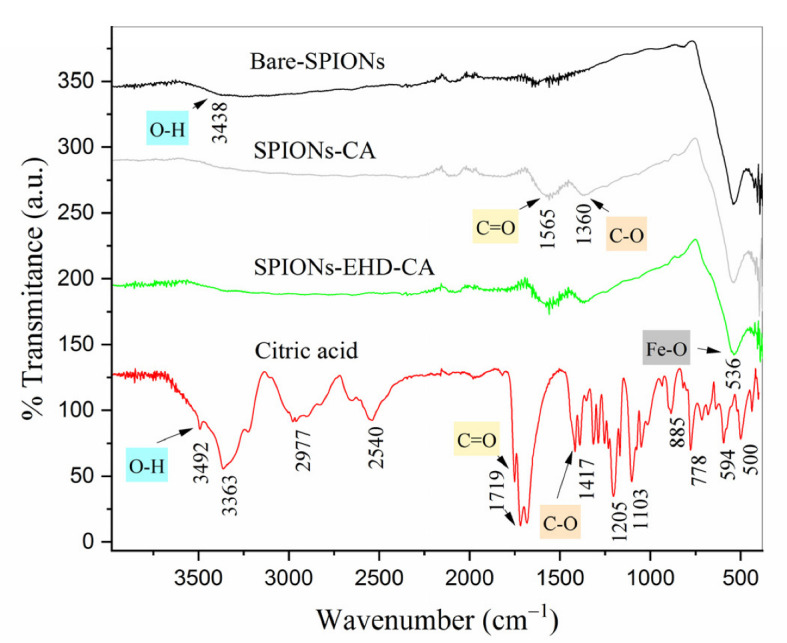
ATR-FTIR spectra of bare magnetite nanoparticles (Fe_3_O_4_), CA-capped SPIONs and pure citric acid. The data are shifted vertically for better visibility.

The synthesized samples have been characterized by FTIR spectroscopy (Figure 4).

The characteristic absorption band of bare magnetic nanoparticles located at 536 cm^−1^ is associated with the stretching vibration mode of Fe–O which is characteristic of iron oxide [54], while the absorption bands at 3438 cm^−1^ of the O–H stretching vibrations indicate the presence of OH groups in the MNPs’ surface. For the CA spectrum, an intense band at 3492 cm^−1^ shows the presence of non-dissociated OH groups. The peak at 1719 cm^−1^ can be assigned to the stretching vibration of C=O group [55,56].

Comparing the spectra of bare and CA-capped samples, it can be seen that several peaks appeared in the 1565 and 1360 cm^−1^ for CA-capped samples (Figure 4) due to the binding of a citric acid radical to the magnetite surface by chemisorption of the carboxylate ions [56].

### 3.4. Ultraviolet-Visible (UV-Vis) Spectroscopy

**Figure 5:**
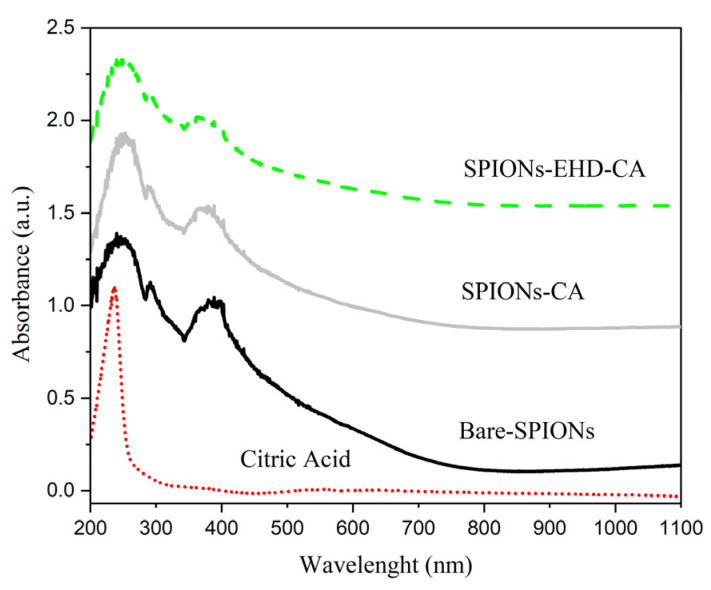
UV-VIS spectra of citric acid solution, bare-SPIONs and CA-capped SPION dispersions. Data are shifted vertically for better visibility.

UV-VIS spectroscopy was used to measure the extinction (scatter plus absorption) of light passing through a sample (Figure 5). Measurements were performed on liquid samples in a quartz cuvette over a spectral range of 200–1100 nm at room temperature.

The citric acid dissolved in distilled water has a strong absorbance band in the UV-region (range 200–260 nm). In this region, bare-SPIONs have also a main adsorption peak at 260 nm and an additional peak in the visible region near 380 nm; this is in agreement with previous reports [57,58]. The influence of CA on the spectra of CA-capped SPIONS is apparent in the steep slopes on both sides of the 240 nm peak, which is uncharacteristic for bare-SPIONs. An apparent shift in the low wavelength peak position of the CA-capped SPIONS can be noticed, which might be related to their less agglomerated morphology compared with that of the bare-SPIONs.

### 3.5. Hydrodynamic Sizes and Zeta Potential Measurements

**Figure 6:**
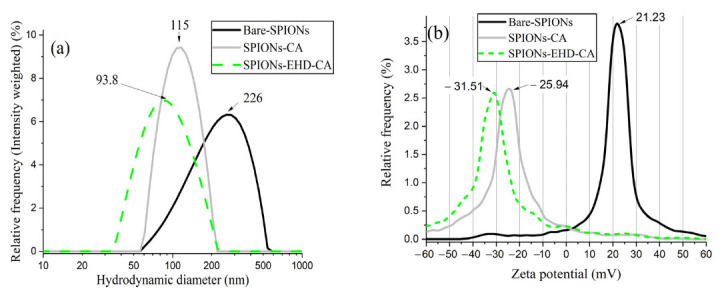
Hydrodynamic size obtained from DLS (**a**) and zeta potentials (**b**).

The characteristic hydrodynamic sizes of the particles and zeta potential (ζ) of the aqueous suspensions are shown in Figure 6.

Dynamic light scattering (Figure 6a) indicates a broad size distribution for the bare-SPIONs with a mean diameter of 226 nm, as the uncoated magnetite nanoparticles tend to aggregate and form clusters with large hydrodynamic sizes. Before CA coating, all the samples were ultrasonicated, which resulted in a narrow size distribution (a mean diameter of 115 nm for sample CA-SPIONs) and smaller diameter. The sample prepared with electrohydraulic discharges displayed an even smaller average cluster size (93.8 nm).

**Figure 7:**
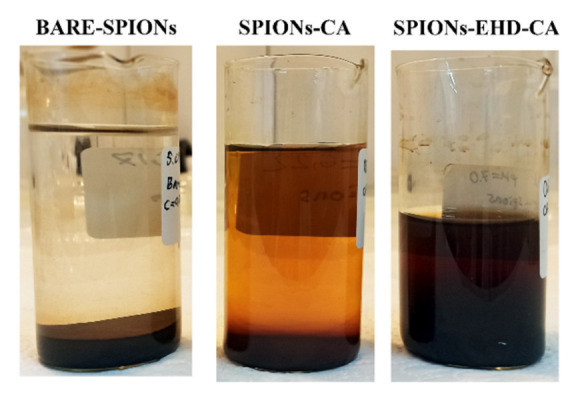
Visual observation of the synthesized samples without centrifugation. The picture is taken 7 months after the synthesis.

For the assessment of the stability of the colloidal dispersions, and thus the strength of electrostatic repulsion between similarly charged particles, the zeta potential (ζ) of the aqueous suspensions was measured (Figure 6b). CA forms a negative charge around magnetite nanoparticle surfaces, while bare-SPIONs are positively charged. The larger negative value of zeta potential (−31.51 mV) of the electrohydraulically processed samples implies that these nanofluids are more stable than the directly CA-capped samples (−25.94 mV). This is also proven by visual observation of the samples after 7 months storage time (Figure 7). According to our previous studies, electrostatic stabilization employing CA coating provides stability for the magnetic fluids in the range of 8–12 month [34].

### 3.6. Small Angle X-ray Scattering (SAXS)

**Figure 8:**
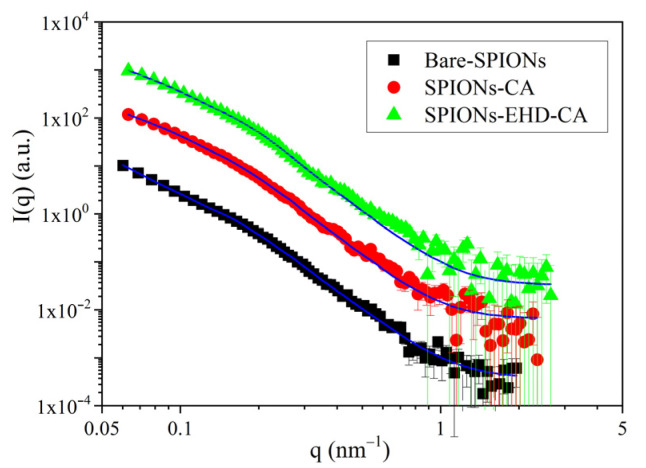
SAXS profiles and fitting by a model of polydisperse spheres using software McSAS. The solid lines are the model fits.

The angular distribution of X-rays scattered by the samples is displayed in Figure 8. in the form of scattering intensity in the function of the scattering vector magnitude q.

**Figure 9:**
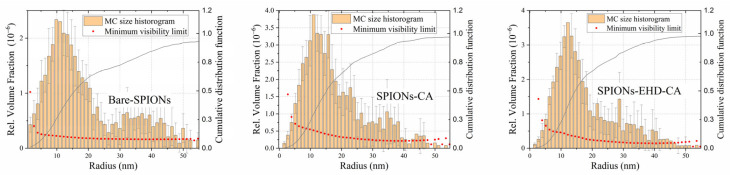
Particle size distributions obtained using McSAS.

After data treatment that included subtraction of scattering of the carrier liquid by software ATSAS v3.2.0 [59], the scattering data were modelled as originating from a broad size distribution of spherical particles using a Monte Carlo fitting routine implemented in software McSAS v1.3.1 [60,61]. The resulting size distributions (Figure 9) include both single and agglomerated SPIONs. The first maximum of the distribution corresponds to the single nanoparticles, with a mean diameter of 25 nm which is in good agreement with the average crystallite size obtained by XRD. It can be seen that for bare-SPIONs (Figure 9a), the size distribution has a prominent second maximum around sizes 35–40 nm which corresponds to a population of large agglomerates. This feature is reduced in the sample with CA-capped particles (Figure 9b), and it is the weakest, as seen in Figure 9c, for the electrohydraulically processed CA-capped particle system.

## 4. Conclusions

Biocompatible IONPs were synthesized using a sonochemical co-precipitation method and then capped with citric acid to render particles with reactive carboxyl groups on their surface and to provide additional functionality for biomedical application. The state of dispersion and aggregation has been influenced by electrohydraulic pretreatment of the magnetite particles before the citric acid capping step. XRD, FTIR and zeta potential measurements revealed the magnetite (Fe_3_O_4_) phase for bare and citric acid functionalized SPIONs and the successful attachment of the functional groups on the particle surface. VSM measurements of the powders revealed higher magnetization of the electrohydraulically processed particles. The saturation magnetization of 61 emu/g and average crystallite and particle diameter ~25–28 nm, as obtained by XRD and SAXS, confers their superparamagnetic nature. The effect of electrohydraulic treatment could be seen as a ~10% decrease of the particle agglomeration and a substantial improvement of the long-term stability of the colloidal dispersion, lasting over 7 months for the presently prepared materials. These properties make the prepared SPIONs suitable candidates for biomedical applications such as cancer therapies, MRI contrast agents, drug transporters and immunotherapy and hyperthermia tools.

## Data Availability

Not applicable.
